# Optimization of Lipid Production by *Schizochytrium limacinum* Biomass Modified with Ethyl Methane Sulfonate and Grown on Waste Glycerol

**DOI:** 10.3390/ijerph19053108

**Published:** 2022-03-06

**Authors:** Szymon Talbierz, Marcin Dębowski, Natalia Kujawska, Joanna Kazimierowicz, Marcin Zieliński

**Affiliations:** 1 InnovaTree Sp. z o.o., 81-451 Gdynia, Poland; szymon.talbierz@innovatree.pl (S.T.); natalia.kujawska@innovatree.pl (N.K.); 2 Department of Environment Engineering, Faculty of Geoengineering, University of Warmia and Mazury in Olsztyn, 10-720 Olsztyn, Poland; marcin.zielinski@uwm.edu.pl; 3 Department of Water Supply and Sewage Systems, Faculty of Civil Engineering and Environmental Sciences, Bialystok University of Technology, 15-351 Bialystok, Poland; j.kazimierowicz@pb.edu.pl

**Keywords:** *Schizochytrium limacinum*, lipids, waste glycerol, ethyl methane sulfonate, optimization, Plackett–Burman design, response surface methodology

## Abstract

One of the most promising avenues of biofuel research relates to using waste as a starting feedstock to produce liquid or gaseous energy carriers. The global production of waste glycerol by the refinery industry is rising year after year. The aim of the present study was to examine the effect of ethyl methane sulfonate (EMS) on the growth rates and intracellular lipid accumulation in heterotrophically-cultured *Schizochytrium limacinum* microalgae, grown on waste glycerol as the carbon source. The strain *S. limacinum* E20, produced by incubating a reference strain in EMS for 20 min, was found to perform the best in terms of producing biomass (0.054 g_DW_/dm^3^·h) and accumulating intracellular bio-oil (0.021 g/dm^3^·h). The selected parameters proved to be optimal for *S. limacinum* E20 biomass growth at the following values: temperature 27.3 °C, glycerol level 249.0 g/dm^3^, oxygen in the culture 26%, and yeast extract concentration 45.0 g/dm^3^. In turn, the optimal values for lipid production in an *S. limacinum* E20 culture were: temperature 24.2 °C, glycerol level 223.0 g/dm^3^, oxygen in the culture 10%, and yeast extract concentration 10.0 g/dm^3^. As the process conditions are different for biomass growth and for intracellular lipid accumulation, it is recommended to use a two-step culture process, which resulted in a lipid synthesis rate of 0.41 g/dm^3^·h.

## 1. Introduction

One of the priorities for researchers, designers, and operators of energy systems is the development and effective deployment of clean energy technologies on an industrial scale. This is motivated, in part, by the need to reduce greenhouse gas emissions, which would directly mitigate the fast-progressing climate change [1,2]. Attempts to deploy biofuel production systems on an industrial scale have shown that such technologies are complex process-wise, difficult to maintain, and require high investment costs [3]. Therefore, new, alternative, and competitive solutions need to be sought—ones which would balance cost-effectiveness with environmental benefits.

One of the most promising avenues of biofuel research relates to using waste as a starting feedstock to produce liquid or gaseous energy carriers [4,5]. The global production of waste glycerol from the refinery industry is rising year after year [6]. There is evidence suggesting that glycerol may be used as a feedstock to grow high lipid-yielding heterotrophic microalgae for biodiesel production [7]. The present study was inspired by the need to effectively manage waste glycerol, combined with the authors’ belief—supported by the current body of research—that glycerol can be used to produce microalgae with a high intracellular content of lipids [8,9].

However, it must be acknowledged that the implementation of efficient and cost-effective microalgae production technologies and adapting them for energy production is problematic due to multiple barriers and limitations [10]. The challenge lies in developing optimized culture protocols, low-cost methods for separation/thickening of biomass, and technologies for extracting the energy carriers [11]. One of the crucial issues is to identify and isolate promoters of microalgal biomass growth, as well as to increase bio-component production [12]. This is currently performed by deploying innovative photobioreactor designs, modifying culture conditions, selecting optimal strains, and applying targeted genetic modifications [13,14]. By comparison, there are little data on the potential use of mutagenic chemicals to tailor the properties of microalgae for biofuel production [15].

The use of ethyl methane sulfonate was determined by its characteristics and proven mutagenic efficacy [16]. It is an organic chemical compound, the use of which causes random point mutations in the genetic material by nucleotide substitution (especially by guanine alkylation). The ethyl group reacts with guanine to form the O-6-ethylguanidine base. During replication, DNA polymerase inserts thymine in place of cytosine and the G:C base pairs become A:T base pairs [17]. EMS is often used as a mutagen in genetic testing [18,19] with its influence on changes in the metabolism of microalgae of such species as *Chlorella vulgaris* [20], *Chlamydomonas reinhardtii* [21], and *Nannochloropsis* sp. [22].

The aim of the present study was to examine the effect of ethyl methane sulfonate on the growth rates and intracellular lipid accumulation in heterotrophically-cultured *Schizochytrium limacinum* microalgae, grown on waste glycerol as the carbon source. The experiment tested the following hypotheses: (1) glycerol is a good source of carbon for heterotrophic culturing in bio-oil-accumulating *Schizochytrium limacinum* microalgae; (2) exposing a reference strain of *Schizochytrium limacinum* to ethyl methane sulfonate enhances biomass growth and intracellular lipid accumulation in heterotrophic, glycerol-grown cultures.

## 2. Materials and Methods

### 2.1. Experimental Design

The experiment involved three stages. During stage 1, the effect of ethyl methane sulfonate (EMS) on *Schizochytrium limacinum* survival rates, biomass growth, and intracellular lipid accumulation was determined, and the best-performing strain was selected. Stage 1 was executed in seven variants, with *Schizochytrium limacinum* being exposed to EMS for different periods of time (V1—0 min (control sample), V2—5 min, V3—10 min, V4—15 min, V5—20 min, V6—25 min, V7—30 min).

Stage 2 assessed how the choice of carbon source impacts biomass growth and lipid accumulation. This stage was divided into three variants according to the organic feedstock used (V1—glucose, V2—crude glycerol, V3—refined glycerol). Afterward, Plackett–Burman and Central Composite Design (CCD) (a form of response surface methodology) were used to optimize the *Schizochytrium limacinum* culture for better productivity, accounting for process parameters and the type of exogenous carbon source.

Stage 3 involved a two-step culture where the strain selected in stage 1 was tested under the optimized process parameters established during stage 2.

### 2.2. Materials

#### 2.2.1. Subsubsection

*Schizochytrium limacinum* (ATCC 20889) was used for the experiment. The strain was stored in the dark on agar slants (medium M1 + 1% agar *w*/*v*, 20 °C) and grown in Erlenmeyer flasks (20 cm^3^, medium M1, 20 °C, 72 h). During the next 48 h, the colonies were agitated on an orbital shaker (100 rpm, 26 °C, New Brunswick Excella E24R, Edison, NJ, USA). The *Schizochytrium limacinum* culture obtained using this process was used as an inoculum for the experiment.

#### 2.2.2. Growth Media and Chemical Reagents

The composition of the growth media used in the experiment is presented in Table 1. All growth media were autoclave-sterilized before use (121 °C, 20 min, Systec VE-95, Systec GmbH, Wettenberg, Germany).

An amount of 0.1 M sodium phosphate buffer (pH 8) was prepared from 4 cm^3^ of a 0.2 M solution (13.9 g of NaH_2_PO_4_ in 500 cm^3^ of dH_2_O) and mixed with 71 cm^3^ of a 0.2 M solution (28.4 g of Na_2_HPO_4_ in 1 dm^3^ of dH_2_O); then made up with dH_2_O to 600 cm^3^, and autoclaved (121 °C; 20 min; Systec VE-95, Systec GmbH, Wettenberg, Germany). The glycerol (CAS 56-81-5) used in the study was sourced from PKN Orlen S.A (glycerol fraction 75.0% *w*/*w*, methanol 12.4% *w*/*w*, moisture 8.3% *w*/*w*, ash 4.3% *w*/*w*, pH 5.6). Soaps, methanol, and free fatty acids (FFAs) were removed from the glycerol for stage 2 by homogenizing the crude glycerol on a mechanical shaker (500 rpm, 5 min, Ika Eurostar 100 control) with demineralized water (HLP 30 UV, Hydrolab, Gdańsk, Poland) in a 1:3 ratio (*v*/*v*) for reduced viscosity. The pH was reduced to 3.0 with hydrochloric acid (at this pH level, soaps transform into free fatty acids, which precipitate). Phase separation was accomplished in a centrifuge (4500 rpm, 10 °C, 20 min, Hettich Universal 320 R, Andreas Heittich GmbH & Co., Tuttlingen, Germany). The FFA-containing phase was decanted out. The resultant refined glycerol was incorporated into the growth media, which were then autoclaved (121 °C, 20 min, Systec VE-95, Systec GmbH, Wettenberg, Germany). Ethyl methane sulfonate (EMS, Sigma-Aldrich, St. Louis, MO, USA) is an organic compound with the molecular formula C_3_H_8_O_3_S. It is a mutagenic ethyl ester of methanesulfonic acid.

### 2.3. Experimental Station

The experiment was conducted in dark bioreactors with a working capacity of 2 dm^3^ (Biostat B Twin, Sartorius Stedim Gmbh, Göttingen, Germany). The reactor was fitted with probes to measure pH, temperature, medium level, foam level, and oxygen concentration. Peristaltic pumps were used to supply pH-stabilizing agents (0.1 M H_2_SO_4_ and 0.1 M NaOH) and an anti-foaming agent (SE-15 Sigma-Aldrich). The stirring system consisted of five-bladed Rushton turbines, bubblers, and four vortex breaks. The culture temperature was maintained constant at 26 °C. Partial oxygen saturation was maintained at 30% by increasing turbine speed from 400 rpm to 800 rpm, or by injecting compressed oxygen gas into the culture (Linde, industrial-grade 99.5% oxygen, 3.5). The culture was aerated to 0.2 vvm. Culture pH was kept at 6.5 ± 0.1. Inoculum volume was 10% (*v*/*v*). After inoculation, the initial dry biomass concentration in the bioreactor was 5.0 g/dm^3^. The culture was aseptically sampled for analyses every 8 h. Growth curves were plotted from the measurements of dry cell weight in the bioreactor. Lipid content was determined gravimetrically.

### 2.4. Experimental Procedure—Stage 1

#### 2.4.1. Treatment of Algae with EMS

The treatment was performed as per the protocol proposed by McCann (2009). To this end, 15 cm^3^ of fresh microalgal culture of cell density 2 × 10^6^/cm^3^ was centrifuged (7000× *g*, 10 min, 10 °C, Hettich Universal 320R, Andreas Heittich GmbH & Co., Tuttlingen, Germany) and suspended in 10 cm^3^ of the phosphate buffer (pH = 8). A total of 100 μL of EMS was then added. The samples were vortexed using a test tube laboratory shaker (Heidolph Reax Top, Heidolph, Germany), then incubated in the dark for 0.5 h at 26 °C. Next, 500-μL samples were taken after 10, 15, 20, 25, and 30 min. Each sample was amended with 500 μL of sodium thiosulfate via a sterile filter (0.2 μL), then shaken for 10 min to inhibit the EMS. After EMS inactivation, the cells were centrifuged (7000× *g*, 10 min, 10 °C, Hettich Universal 320 R, Andreas Heittich GmbH & Co., Tuttlingen, Germany), and resuspended in a fresh, sterile M1 medium. The bon-EMS-treated culture served as the control.

#### 2.4.2. Incubation

All EMS-treated samples were diluted 10^−1^, 10^−2^, 10^−3^. In total, 25 μL of each sample was transferred onto sterile Petri dishes with agar-solidified (1% *w*/*v*) medium M1. The plates were incubated in the dark at 26 °C until microalgal colonies emerged, after which survival diagrams were constructed. All microalgal colonies were transferred to the tubes with 5 cm^3^ of the agar-solidified M1 growth medium (1% *w*/*v*) in slants and placed in a cooler (6 °C). Each colony was assigned a specific designation, where: E means “EMS-treated” and the number means the exposure time.

#### 2.4.3. Microalgae Screening

Individual microalgal colonies were first checked for their capacity to grow in a glycerol-based liquid medium. To this end, 20-μL samples of the microalgal cultures were placed in a 96-well microplate. A total of 180 μL of the M3 growth medium was added to each well. The resultant plates were incubated in the dark in an orbital shaker (temp 26 °C, 180 rpm, New Brunswick Excella E24 R, Edison, NJ, USA) for 72 h. In total, 20 μL of the culture was transferred to new microplates with 180 μL of the M3 medium. The plates were incubated under the same conditions for 7 days. Every 12 h, the optical density of the culture was measured using a spectrophotometer (Multiskan GO microplate reader, ThermoFisher Scientific, Vartaa, Finland). A growth curve DW = f(t) was plotted as well as the growth rate r_DW_ determined for the fastest-growing strain.

In the second round of screening, the strains were selected for the highest lipid accumulation rates (r_LIP_). Only strains that passed the first round of screening were included. As before, the plates were stirred post-incubation (7000× *g*, 10 min, 10 °C, Hettich Universal 320 R). The supernatant was removed and 180 μL of the sterile M2 growth medium was added to the concentrated biomass (the medium was nitrogen deprived to induce lipid accumulation). The cultures were placed in the dark in an orbital shaker (26 °C, 180 rpm, 48 h, New Brunswick Excella E24 R, Edison, NJ, USA). The lipid levels were then determined. Survival rates were determined for all strains, calculated as the ratio between the colony count after incubation in EMS and the non-incubated control (%). Selected strains were characterized by comparing the biomass growth rate and lipid accumulation to the reference strain after being grown in bioreactors (Biostat B Twin, Sartorius Stedim Gmbh, Göttingen, Germany).

### 2.5. Optimization Design—Stage 2

Multiple carbon sources were assessed for their effect on *S. limacinum* E20 biomass growth and intracellular lipids. Growth media designated M5, M6, and M7 were used (Table 1). The cultures were grown in bioreactors (Biostat B Twin, Sartorius Stedim Gmbh, Göttingen, Germany).

#### 2.5.1. Plackett–Burman Design

Plackett–Burman design was employed in the glycerol variant to identify the physical and chemical culture parameters most influential to cell growth rate, final biomass levels, and intracellular lipid levels. Ten variables were screened with the design at two levels—low (−) or high (+) (Table 2)—in 12 culture variants (Table 3). Parameters were assigned their levels (high/low) based on preliminary research and the literature data. The final effect of each variable (E) was calculated from the equation

$$E_{\left(\mathrm{xi}\right)}=\frac{2*\left(\sum M_{i+}-\sum M_{i-}\right)}{N}$$

where: E_(xi)_—effect of the variable, M_i+_—dry weight or lipid concentration for the high (+) level of the variable, M_i−_—dry weight or lipid concentration for the low (**−**) level of the variable, N—number of runs.

The resultant effects of the dummy variable (d1) were considered to be the standard error of the experiments, which could be used to derive the significant level (Wen and Chen 2001). Design-Expert software (Stat-Ease Inc., Minneapolis, MN, USA) was used to produce the variable table and conduct the entire statistical analysis (*F-*test). Variables had to be *p* < 0.10 to be considered significant for the DM and lipid concentrations in the culture.

#### 2.5.2. Central Composite Design (CCD)

The culture parameters screened with the Plackett–Burman method were further optimized using CCD. A matrix of 30 runs was generated, with six central and eight axial points. Only one variable was set at extreme (−2 or +2) level, with the remainder being at the central point. The variables are presented in Table 4.

Based on the experimental results, a second-order polynomial equation was derived to express the production of the dry microalgal cell weight and the lipid production as a function of independent variables:

$$Y={\;Z}_{0}+\sum {\;Z}_{i}x_{i}+\sum {\;Z}_{i}x_{i}^{2}+\sum {\;Z}_{\mathrm{ij}}x_{i}x_{j}$$

where: Y—predicted response of the design, Z—coefficients of the equation, x_i_ and x_j_—coded levels of the variables.

Those other parameters that had been deemed non-significant were permanently set as low. The process was used to determine the optimal culture conditions for maximizing culture performance and lipid production in a single-phase batch culture of microalgae.

#### 2.5.3. Validation of Optimal Culture Conditions

A microalgae culture was set up to verify the optimal process parameters. In the first variant, the parameter values were selected for high production of microalgal biomass, in the second—for maximum lipid concentration in the biomass. Dry cell weight, glycerol in the growth medium, and lipid content of the microalgal biomass were monitored throughout the culture process.

### 2.6. Analytical Methods

The optical density was measured spectrophotometrically (Multiskan GO, ThermoFisher Scientific, Vartaa, Finland) at a wavelength of 550 nm [20]. Dry cell weight was derived from the calibration curve (g_DW_/dm^3^) = f(OD_550_), R^2^ = 0.9984. The protocol by Huang et al. (2009) was used to determine the lipid content [23]. The freeze-dried microalgal biomass was diluted with 150 μL of distilled water and 20 μL of the Nile red solution in DMSO (0.5 × 10^−3^ mg/cm^3^). The samples were vortexed using a test tube laboratory shaker (Heidolph, Reax top). Fluorescence was measured with a spectrofluorometer (HITACHI F-4500, Hitachi, Tokyo, Japan) with a 480 nm excitation filter and 570–590 nm emission filters. Fluorescence intensity correlates linearly with the gravimetrically-determined lipid content (R^2^ = 0.9945). For the gravimetric quantification of lipids, 1 g of the freeze-dried microalgal biomass was mixed with 5 cm^3^ of chloroform, 5 cm^3^ of methanol, and 1 cm^3^ of a 5% NaCl solution. Suspended microalgae were vortexed at a high speed on a test tube laboratory shaker (Heidolph Reax Top, Heidolph, Germany) for 2 min, then centrifuged (8000× *g*, 4 min, 10 °C). The chloroform layer was collected. The process was repeated three times and the collected chloroform mixed together. Chloroform was then distilled out with a rotary evaporator, and the resultant lipid residue was weighed. To determine dry weight, a culture sample was centrifuged (4000× *g*, 5 min, 10 °C). The concentrated biomass was washed with distilled water and dried in a freeze dryer to the constant weight. This sample was then weighed on a micro-analytical balance. Fatty acid methyl esters (FAMEs) were prepared using the previously freeze-dried microalgal biomass which was transesterified according to Cohen et al. (1997) [24]. The resultant methyl esters were extracted with hexane. A Clarus 680 GC (Perkin Elmer, Waltham, MA, USA) gas chromatograph was used for ester separation; nitrogen was used as the carrier gas. The initial temperature of the column was 170 °C; it was gradually increased to 225 °C at a rate of 1 °C min^−1^. The other parameters were: injector temperature 250 °C, injection speed 3 μL (splitless mode), FID temperature 270 °C. Qualitative analysis was based on residence time, whereas the area under the peak was used as the indicator for quantitative analysis. Glucose in the culture was determined with a spectrophotometer. To that end, the sample from the bioreactor was centrifuged (8000× *g*, 4 min, 10 °C) and the supernatant was filtered (pore size = 0.2 μm). Three cm^3^ of the filtrate was added to 3 cm^3^ of a 1% DNS solution (10 g of 3,5-dinitrosalicylic acid, 2 g of phenol, 0.5 g of sodium sulfite, 10 g of sodium hydroxide, made up with distilled water to 1 dm^3^) and heated in a water bath to 90 °C for 5–15 min until a reddish brown coloration was obtained. One cm^3^ of potassium sodium tartrate was added to stabilize the color. After the mixture was cooled to room temperature, absorbance was measured (575 nm) using a spectrophotometer (Multiskan GO, ThermoFisher Scientific, Vartaa, Finland). Glucose concentration was read from the calibration curve. Glycerol concentration in the growth medium was determined using the standard (enzymatic) method with a Glycerol GK Assay Kit (Megazyme, Bray, Ireland). The determination was made by centrifuging a sample from the bioreactor (8000× *g*, 4 min, 10 °C), filtering the supernatant (pore size = 0.2 μm), and assaying the filtrate for glycerol levels using the kit. The test involved first phosphorylating the glycerol with adenosine 5′-triphosphate (ATP). The reaction product—adenosine 5′-diphosphate (ADP)—was then used to further phosphorylate d-glucose, which oxidizes while producing nicotinamide adenine dinucleotide (NADH). Afterward, NADH was quantified spectrophotometrically at 340 nm. To determine residue on ignition, 3 g of glycerol were placed in heat-resistant crucibles (which had been dried for 3 h at 120 °C and weighed) and heated at 750 °C for 3 h. The resultant residue was cooled to room temperature in a desiccator and weighed.

### 2.7. Statistical Analysis

The statistical analysis of the experimental results was conducted using STATISTICA 13.3 PL. One-way analysis of variance (ANOVA) was used to determine significant differences across the groups. Significant differences between the variables were determined via Tukey’s HSD test (*p* = 0.05). The analyses were performed in triplicate.

## 3. Results and Discussion

### 3.1. Stage 1

During the course of this study, a library of 62 individual colonies was generated. EMS treatment was found to have a detrimental effect on *Schizochytrium limacinum* survival, with survivability decreasing with longer exposure times. After only 10 min of incubation, the survival threshold dropped to less than 20%. *S. limacinum* E15 was the fastest-growing strain, with the r_DW_ reaching 0.059 ± 0.002 g_DW_/dm^3^·h. When adjusting for biomass growth and lipid accumulation efficiency, *S. limacinum* E20 proved to be the most efficient lipid producer, accumulating lipids at a rate of 0.021 ± 0.03 g lipids/dm^3^·h during incubation. The r_DW_ for *S. limacinum* E20 was 0.054 ± 0.004 g_DW_/dm^3^·h (Table 5).

**Figure 1:**
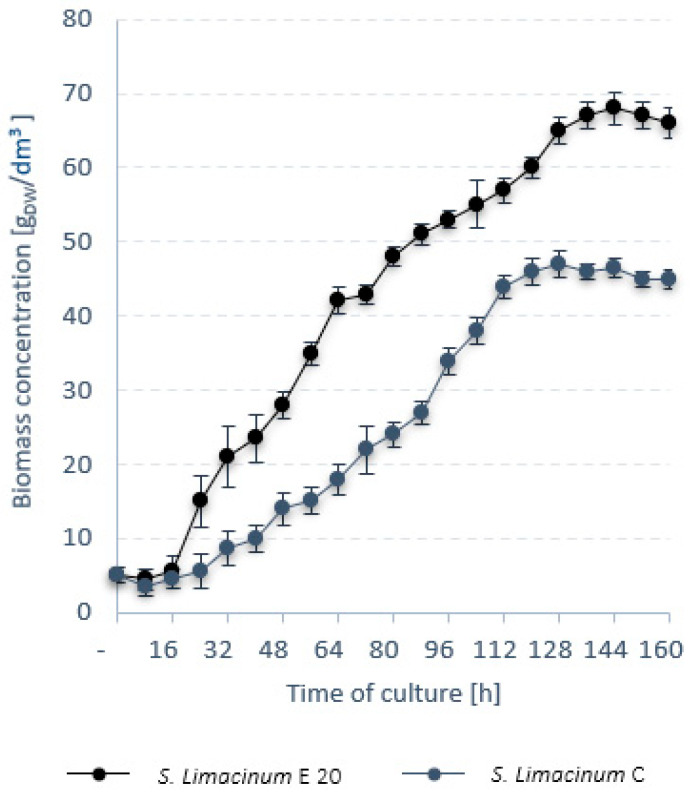
Trends in microalgal biomass.

**Figure 2:**
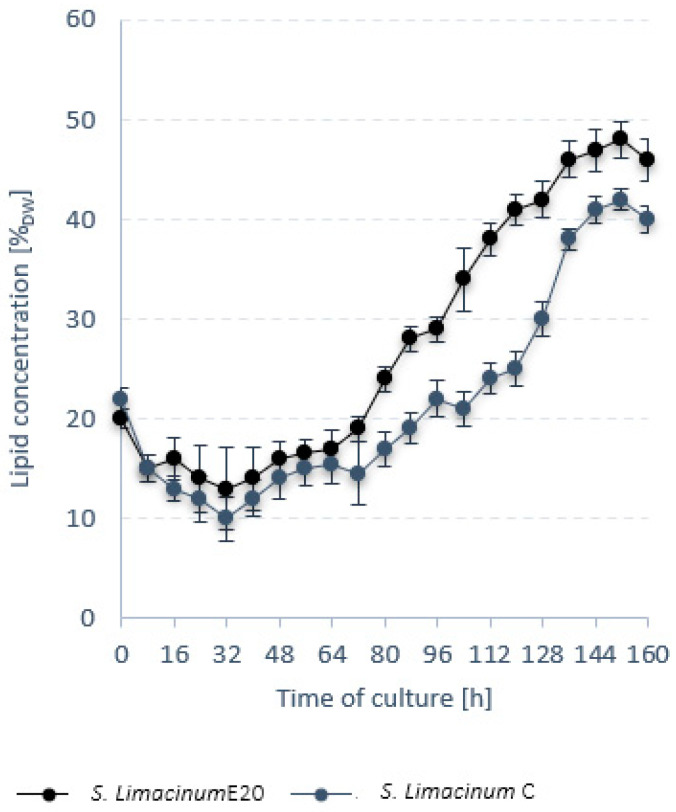
Trends in lipid content of the microalgal biomass.

Further into stage 1, a comparative analysis was performed between *S. limacinum* E20 and the reference strain *S. limacinum* C. *S. limacinum* E20 biomass ceased to grow after 144 h of culture. For *S. limacinum* C*,* the growth stage lasted 128 h (Figure 1). The final biomass concentration in the bioreactor for *S. limacinum* E20 was 68.0 ± 0.3 g_DW_/dm^3^ and was significantly higher than for *S. limacinum* C, which only grew to 47.0 ± 0.4 g_DW_/dm^3^ (Figure 1). In both cases, the lipid content of the biomass peaked at 152 h of culture (Figure 2). The lipid fraction of *S. limacinum* E20 was determined to be 48 ± 1.2% dry weight at r_LIP_ = 0.21 ± 0.03 g/dm^3^·h, whereas *S. limacinum* C produced 42 ± 0.9% dry weight and r_LIP_ = 0.12 g/dm^3^·h (Table 6). The essential composition of the biomass is presented in Table 6. *S. limacinum* C had higher protein and carbohydrate fractions (by 7% and 1%, respectively). Both strains were mainly composed of saturated fatty acids, especially palmitic acid (C16:0), which accounted for 61.02 ± 0.4% in *S. limacinum* E20 and 54.24 ± 0.4% in *S. limacinum* C. High concentrations were also recorded for docosahexaenoic acid (DHA, 22:6), at 26.24 ± 1.1% for *S. limacinum* E20, and 31.23 ± 0.7% for *S. limacinum* C (Table 6).

Zhang et al. (2016) also observed a positive effect of EMS on the improvement of the obtained biomass yield and lipid productivity by desert microalgae *Desmodesmus* sp. strains S81 and G41. In the case of *Desmodesmus* sp. S81, the best results were obtained after a 60-min exposure to 0.6 M EMS. The production of biomass was 778.10 mg/dm^3^, the lipid content of 48.41%, and the concentration of lipids 19.83 mg/dm^3^·d. These values were 45.50%, 8.00%, and 74.24%, respectively, higher than the cultivation without EMS. The optimal conditions for exposure to EMS for *Desmodesmus* sp. G41 were 30 min in 0.8 M EMS concentration. The obtained biomass concentration was 739.52 mg/dm^3^, lipid content 46.01%, and lipid concentration at the level of 17.92 mg/dm^3^·d. The analyzed indicators increased by 20.67%, 10.35%, and 55.77%, respectively, compared to *Desmodesmus* sp. G41 not treated with the mutagenic factor [25]. Tanadul et al. (2018) also obtained promising results [20]. After 30-min exposure to 100–200 mM EMS, the biomass concentration was 640 mg/dm^3^, which was a 111% increase in relation to the reference sample. The lipid concentration increased by 59% [26]. The positive effect of EMS on the biomass of microalgae was also reported by other researchers [27,28,29].

### 3.2. Stage 2

**Figure 3:**
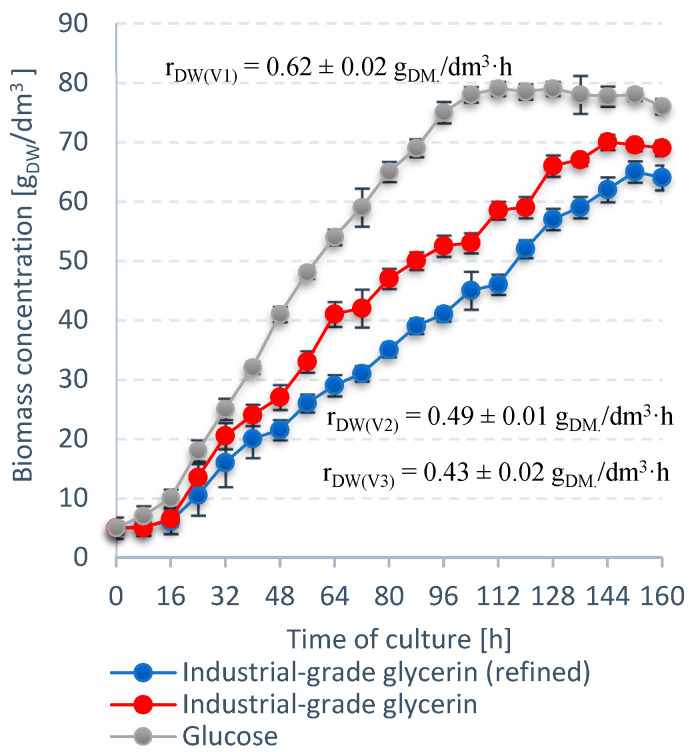
Trends in biomass concentration for *S. limacinum* E20 biomass for different carbon sources.

**Figure 4:**
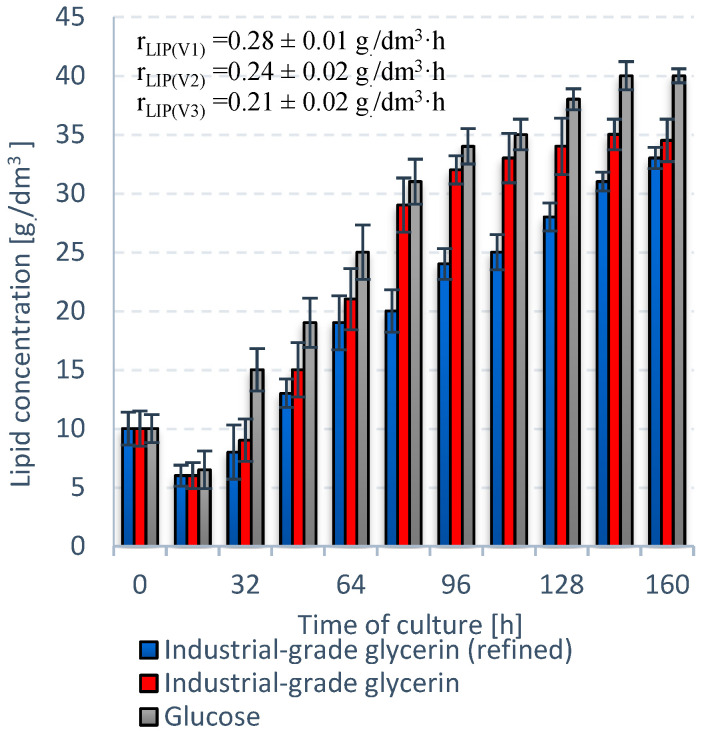
Trends in lipid content for *S. limacinum* E20 for different carbon sources.

V1 exhibited the best performance in terms of *S. limacinum* E20 biomass growth (Figure 3). The culture entered the stationary growth phase at 128 h with a biomass level of 79 ± 0.2 g_DW_/dm^3^. Crude glycerol (V2) proved to be a more suitable carbon source than refined glycerol (V3), with a difference of almost 5 g_DW_/dm^3^ (Figure 3). The V2 culture was also quicker to reach stationary growth phase at 144 h. V1 was the most productive variant in terms of lipids. The lipid fraction in the V1 biomass reached a maximum of 51 ± 1% dry weight and r_LIP_ of 0.28 ± 0.01 g/dm^3^·h (Figure 4). The lipid concentration—35.0 ± 0.3 g/dm^3^—was higher in V2 than V3 (33.0 ± 0.2 g/dm^3^) (Figure 4).

The highest r_DW_ and r_LIP_ were recorded for V1, reaching 0.62 ± 0.02 g_DW_/dm^3^·h and 0.28 ± 0.01 g/dm^3^·h, respectively (Figure 3 and Figure 4). V2 produced higher rates of r_DW_ and r_LIP_ than the V3, with differences of ∆r_DW_ = 0.06 and ∆r_LIP_ = 0.03. Moreover, the dry weight (70.0 ± 0.1 g_DW_/dm^3^) and lipid concentration (35.0 ± 0.3 g/dm^3^) were higher in this variant (Figure 3 and Figure 4).

The *S. limacinum* strain is capable of growing and of lipid accumulation on various carbon sources. Yokochi et al. (1998) confirmed the ability of this microalgae to grow on nine different carbon sources, including glucose, fructose, and oleic acid. The highest biomass concentration, 16 g_DM_/dm^3^ was obtained for oleic acid, while the highest DHA content 1.1 g/dm^3^, was observed for the glycerol-based medium. [30]. The cost of the carbon source such as glucose, is high compared to other nutrients (nitrogen, micronutrients) that must be added to the microalgae culture [31]. Thus, in order to increase the economic efficiency of the heterotrophic bio-oil production technology using microalgae, other, cheaper sources of carbon should be used. One of the proposed solutions is waste technical glycerin, which allows a high final concentration of microalgae biomass and desired bioproducts to be obtained [32,33]. Rattanapoltee et al. (2021) confirmed the marginal influence of impurities in raw technical glycerin, e.g., soap, methanol, on the final concentration of biomass and the final content of valuable fermentation products such as DHA [34]. According to Pyle et al. (2008), during the process of thermal sterilization (autoclaving at 121° C, 20 min), methanol is removed from the nutrient solution based on technical glycerol [35].

The *S. limacinum* E20 culturing process was then optimized for biomass and lipid production by using crude glycerol. Twelve culture variants were screened using Plackett–Burman design (Table 7).

The results were used to calculate the effect of each tested variable and their corresponding F and *p* values (Table 8). The standard error was derived from the effect calculated for the dummy variable (the variable designated d1), which was 3.23 g_DW_/dm^3^ for the dry cell weight in the culture and 1.70 g/dm^3^ for the lipid concentration. Variables with effects at *p* < 0.10 were considered statistically significant. It was found that out of the 10 tested physical/chemical parameters (Table 2), the temperature (°C), glycerol level (g/dm^3^), oxygen in the culture (% saturation), and yeast extract concentration (g/dm^3^) had a significant impact on *S. limacinum* E20 culture productivity. Accordingly, they were selected for further optimization using CCD. The experimental design matrix consisted of 30 runs, with four significant variables screened at five levels (−2, −1, 0, 1, 2). The values for the coded variables are presented in Table 9.

Runs 25–30, whose variables were associated to the central points, were performed to determine the standard error for the design, which was 0.74 g_DW_/dm^3^ for the dry cell weight in the culture and 0.56 g/dm^3^ for the lipid concentration. The results of the runs were correlated using Design-Expert and modeled as a function of four independent variables in the form of a second-order polynomial equation. The coefficient values and the corresponding *F* and *p*-values are given in Table 10.

The values of the four tested variables selected as optimal for dry weight production in the system were: temperature 27.3 °C, glycerol level 249.0 g/dm^3^, oxygen in the culture 26%, and yeast extract concentration 45.0 g/dm^3^. When the same parameters were optimized for intracellular lipid accumulation, the values were: temperature 24.2 °C, glycerol level 223.0 g/dm^3^, oxygen in the culture 10%, and yeast extract concentration 10.0 g/dm^3^. Design-Expert was used for the screening. The second-order polynomial equation used to express production of the dry microalgal cell weight was as follows

$$DW\left(\frac{g_{\mathrm{DW}}}{{\mathrm{dm}}^{3}}\right)=81.61+2.588\times Z_{1}+0.896\times Z_{2}+0.229\times Z_{3}+0.154\times Z_{4}$$

The second-order polynomial equation used to express lipid production efficiency (based on the experiments) was as follows

$$LIP\left(\frac{g_{.}}{{\mathrm{dm}}^{3}}\right)=48.03+0.771\times Z_{1}+1.688\times Z_{2}+0.363\times Z_{3}+0.446\times Z_{4}$$

**Figure 5:**
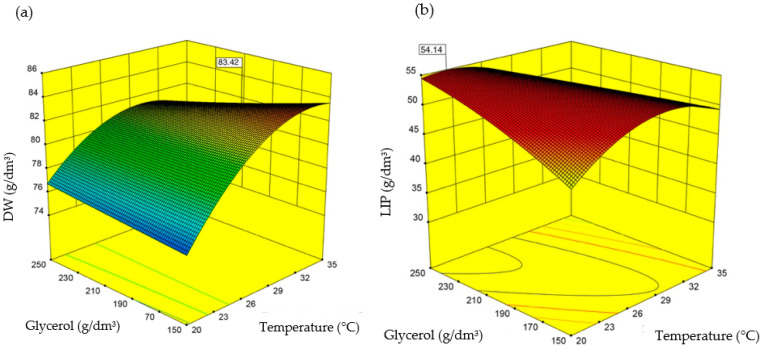
*S. limacinum* E20 dry weight (**a**) and lipids in the biomass (**b**) as a function of temperature and glycerol levels.

The results indicate that introducing more glycerol leads to higher biomass growth and lipid production. Cooler temperatures, lower oxygen, and lower nitrogen in the culture promote intracellular lipid accumulation. The effect of the tested variables on microalgal biomass growth and lipid accumulation rates is presented in Figure 5.

The optimal values of the independent variables, screened using CCD, were verified through laboratory experiments in two separate cultures. The first was grown in conditions optimized for biomass growth, the second—for lipid production. The results were similar to those produced by the mathematical model (Table 11).

**Figure 6:**
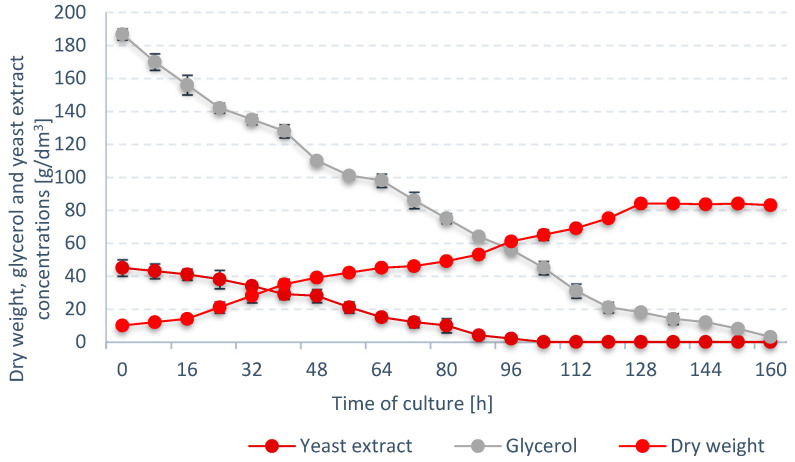
Trends in dry weight, glycerol, and yeast extract concentrations in the batch culture optimized for *S. limacinum* E20 growth.

*S. limacinum* E20 was found to reach the stationary growth phase at 128 h of culture with a final biomass concentration of 84 ± 0.11 g/dm^3^ (Figure 6). Glycerol level in the culture gradually dropped, reaching 3.0 ± 0.1 g/dm^3^ after 160 h of culture. Chang et al. (2013) proved that high glycerin concentrations above 150 g/dm^3^ allow the final dry biomass contraction of *S. limacinum* SR21 to be obtained at the level of 60.71 ± 2.0 g_DW_/dm^3^ [36]. It is also worth mentioning that in the case of some species of microalgae, e.g., *Aurantiochytrium limacinum* SR21, the initial concentration of glycerin in the medium above 100 g/dm^3^ inhibited the culture proliferation [37].

Nitrogen in the medium was also found to steadily decrease, and was finally exhausted after 104 h of bioreactor operation. It was determined that nitrogen take-up had no direct inhibitory effect on the growth of *S. limacinum* E20 biomass—dry cell weight continued to rise throughout the subsequent 24 h of operation (Figure 6). These results correspond to the studies by Yeh and Chang (2012) with the *Chlorella vulgaris* ESP-31 strain using [38]. The efficiency of microalgae cultivation under photoautotrophic, mixotrophic, and heterotrophic conditions was compared. Regardless of the cultivation method, the use of a medium with a low nitrogen content promoted the process of lipid accumulation in microalgae cells, and their final concentration in dry matter ranged from 20% to 53%. Whereas Hu et al. (2008) proved that the composition of fatty acids changes as a result of a decrease in nitrogen concentration in culture [39].

### 3.3. Stage 3

**Figure 7:**
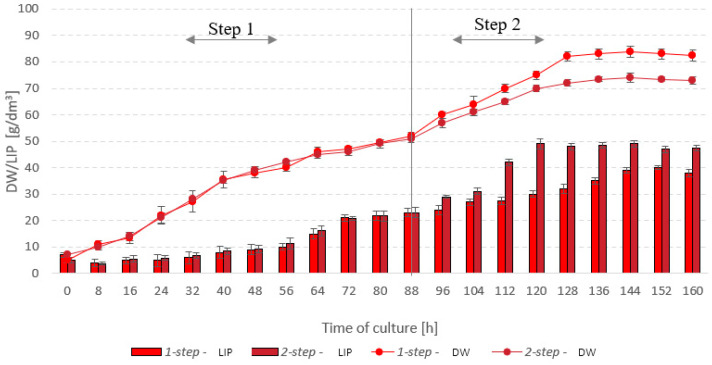
Trends in microalgal biomass and lipid concentrations—one-step vs two-step culture.

Based on the results from stages 1 and 2, it was decided that a two-step culture needed to be employed, with the first step providing optimal conditions for the growth of *S. limacinum* E20 biomass, and the second adapted for rapid lipid accumulation. In this two-step culture, the parameters were switched at 88 h: the temperature was lowered from 27.3 °C to 24.2 °C, and oxygen saturation—from 26% to 10%. The final yields of microalgal biomass were lower in the two-step process—the one-step culture produced a final 84 g_DW_/dm^3^, whereas the two-step reached only 74 g_DW_/dm^3^ (Figure 7). The peak lipid fraction—49 g/dm^3^—was observed at 120 h of culture, which corresponded to a r_LIP_ of 0.41 g/dm^3^·h. In the one-step microalgal culture, lipids were produced at a rate of 0.34 g/dm^3^·h. The lipid concentration in microalgal cells was around 39 g/dm^3^. It was noted that the two-step culture was quicker to reach the stationary phase in terms of the lipid fraction growth. Inhibition of lipid growth in the system was found to occur as early as after 120 h of culture. The same was observed after 144 h for the one-step process (Figure 7).

The use of a two-stage cultivation system is common in the case of microalgae biomass production stimulated to accumulate significant amounts of lipids. In this case, the first stage of the culture is the phase of intensive growth and cell division, in which the culture density increases rapidly, while the amount of lipids in the biomass is low. The second stage of culture is the stage of biosynthesis of the desired product, e.g., lipids, in which the number of microalgae cells slightly increases, while the cells increase their volume and there is an intensive accumulation of oil in the cells [40].

Only the first stage of culture is highly demanded for oxygen due to the fact that rapidly dividing cells require a large number of primary metabolites, i.e., enzymes, nucleic acids, proteins. In turn, in the second phase of cultivation, low oxygen concentration causes increased lipid accumulation [41]. Chi et al. (2009) found that the oxygen concentration above 50% in the last phase of cultivation has an adverse effect on the lipid production efficiency. These observations concerned the heterotrophic culture of *Schizochytrium limacinum* SR21 [40].

## 4. Conclusions

The highest biomass growth rate (0.054 g_DW_/dm^3^·h) and intracellular bio-oil accumulation rate (0.021 g/dm^3^·h) were produced by the strain *S. limacinum* E20, which was obtained by incubating the reference strain in EMS for 20 min. The maximum performance in terms of dry weight production rate and lipid accumulation was achieved when glucose was used as the exogenous carbon source. Out of the other two feedstocks, waste glycerol was found to work better than refined glycerol.

The culture parameters identified as significant drivers of *S. limacinum* E20 biomass proliferation and lipid fraction growth were: temperature, glycerol level, oxygen saturation, and yeast extract concentration in the medium. The parameter values most conducive to *S. limacinum* E20 biomass growth were: temperature 27.3 °C, glycerol level 249.0 g/dm^3^, oxygen in the culture 26%, and yeast extract concentration 45.0 g/dm^3^. Conversely, the optimal values for lipid production in an *S. limacinum* E20 culture were: temperature 24.2 °C, glycerol level 223.0 g/dm^3^, oxygen in the culture 10%, and yeast extract concentration 10.0 g/dm^3^.

Since the optimal culture conditions vary depending on which type of final effect is to be maximized (biomass growth in the system vs. intracellular lipid accumulation), it is recommended to use a two-step culture process, which resulted in a lipid synthesis rate of 0.41 g/dm^3^·h.

## Figures and Tables

**Table 1:** Composition of the growth media used in the experiment.

Medium (Symbol)	Yeast Extract (g/dm^3^)	Peptone (g/dm^3^)	Glucose (g/dm^3^)	Glycerol (g/dm^3^)	Refined Glycerol (g/dm^3^)	Sea Water Made up to the Target vol. (dm^3^)
M1	5.0	5.0	15.0	-	-	1.0 (35 PSU)
M2	0.5	-	-	15.0	-	1.0 (35 PSU)
M3	5.0	5.0	-	15.0	-	1.0 (35 PSU)
M4	20.0	20.0	-	100.0	-	1.0 (35 PSU)
M5	20.0	20.0	-	-	204.4	1.0 (35 PSU)
M6	20.0	20.0	-	136.3	-	1.0 (35 PSU)
M7	20.0	20.0	100	-	-	1.0 (35 PSU)
M8	45.0	-	-	249.0	-	1.0 (15 PSU)

**Table 2:** Variables screened using Plackett–Burman design.

Code	Variable	Low (−)	High (+)
A	Initial cell concentrations in the culture (g_DW_/dm^3^)	5	10
B	Volumetric air flow rate (dm^3^/min.)	0.1	0.5
C	pH	6	8
D	Glycerol level (g/dm^3^)	100	200
E	Culture volume (dm^3^)	1	2
F	Salinity (PSU)	15	35
G	Turbine speed (rpm)	400	800
H	Concentration of yeast extract (g/dm^3^)	10	30
I	Temperature (°C)	20	30
J	Oxygen concentration (%)	20	35

**Table 3:** Variable combinations of the Plackett–Burman design.

Variant No.	A	B	C	D	E	F	G	H	I	J	d1
1	+	+	*−*	*−*	+	+	+	+	*−*	+	*−*
2	*−*	+	+	*−*	*−*	+	+	+	+	*−*	+
3	+	*−*	+	+	*−*	*−*	+	+	+	+	*−*
4	*−*	+	*−*	+	+	*−*	*−*	+	+	+	+
5	+	*−*	+	*−*	+	+	*−*	*−*	+	+	+
6	+	+	*−*	+	*−*	+	+	*−*	*−*	+	+
7	+	+	+	*−*	+	*−*	+	+	*−*	*−*	+
8	+	+	+	+	*−*	+	*−*	+	+	*−*	*−*
9	*−*	+	+	+	+	*−*	+	*−*	+	+	*−*
10	*−*	*−*	+	+	+	+	*−*	+	*−*	+	+
11	+	*−*	*−*	+	+	+	+	*−*	+	*−*	+
12	*−*	*−*	*−*	*−*	*−*	*−*	*−*	*−*	*−*	*−*	*−*

**Table 4:** Values of the independent variables used for the CCD.

Variable	Variable Code	Unit	−2	−1	0	1	2
Temperature	Z_1_	°C	15	20	25	30	35
Glycerol level	Z_2_	g/dm^3^	50	100	150	200	250
Concentration of yeast extract	Z_4_	g/dm^3^	10	30	50	70	90
Oxygen saturation	Z_3_	%	2.5	15	27.5	40	52.5

**Table 5:** *S. limacinum* survival, biomass growth, and lipid accumulation rates vs EMS exposure time.

Strain Name	Variant	Survival Rate (%)	r_DW_ (g/dm^3^·h)	r_LIP_ (g/dm^3^·h)
*S. limacinum* C	V1–reference strain	100	0.046 ± 0.002	0.017 ± 0.02
*S. limacinum* E5	V2–EMS (5 min)	23 ± 4	0.051 ± 0.003	0.017 ± 0.02
*S. limacinum* E10	V3–EMS (10 min)	17 ± 7	0.045 ± 0.005	0.011 ± 0.01
*S. limacinum* E15	V4–EMS (15 min)	16 ± 3	0.059 ± 0.002	0.018 ± 0.02
*S. limacinum* E20	V5–EMS (20 min)	11 ± 2	0.054 ± 0.004	0.021 ± 0.03
*S. limacinum* E25	V6–EMS (25 min)	12 ± 5	0.039 ± 0.002	0.011 ± 0.02
*S. limacinum E30*	V7–EMS (30 min)	2 ± 1	0.031 ± 0.001	0.011 ± 0.03

**Table 6:** Profile of the microalgal biomass.

	Unit	*S. limacinum* E20	*S. limacinum* C
Dry weight	g_DW_/dm^3^	68.0 ± 0.3	47.0 ± 0.4
r_DW_	g_DW_/dm^3^·h	0.47 ± 0.1	0.36 ± 0.1
r_LIP_	g/dm^3^·h	0.21 ± 0.2	0.12 ± 0.1
Lipids	% DW	48 ± 1.2	42 ± 0.9
Proteins	% DW	15 ± 0.5	22 ± 0.8
Carbohydrates	% DW	22 ± 0.3	23 ± 0.5
Ash	% DW	15 ± 0.3	13 ± 0.2
C14:0	% SCFA	3.03 ± 0.5	2.19 ± 0.6
C16:0	% SCFA	61.02 ± 0.4	54.24 ± 0.4
C18:0	% SCFA	3.33 ± 0.3	2.87 ± 0.5
C22:5	% SCFA	5.23 ± 0.4	10.29 ± 0.9
C22:6	% SCFA	26.24 ± 1.1	31.23 ± 0.7

**Table 7:** Biomass and lipid concentration in the *S. limacinum* E20 culture as a function of the tested parameters.

Culture	no.	1	2	3	4	5	6	7	8	9	10	11	12
Dry weight	g_DW_/dm^3^	72.2	73.1	75.2	74.3	68.3	31.8	22.3	73.4	75.1	52.0	67.5	74.0
Lipids	g/dm^3^	37.3	30.0	41.4	39.0	28.0	22.3	21.0	38.5	40.0	25.0	39.1	37.0

**Table 8:** Effect of the tested variable on the growth of dry cell weight and lipids in S. limacinum E20 culture, with the corresponding statistical tests.

	Variable									
**Dry Cell Weight**	**A**	**B**	**C**	**D**	**E**	**F**	**G**	**H**	**I**	**J**
Effect	10.37	14.20	19.93	23.23	17.37	19.57	12.53	20.97	42.43	23.10
F value	10.28	19.29	38.01	51.63	28.85	36.62	15.03	42.05	172.23	51.04
*p* level	0.192	0.143	0.102	0.088	0.117	0.104	0.161	0.097	0.048	0.089
**Lipid Concentration**	**A**	**B**	**C**	**D**	**E**	**F**	**G**	**H**	**I**	**J**
Effect	9.43	9.60	8.20	15.33	10.03	6.97	10.60	10.97	18.90	11.23
F value	30.79	31.89	23.27	81.35	34.83	16.79	38.88	41.62	123.60	43.66
*p* level	0.114	0.112	0.130	0.070	0.107	0.152	0.101	0.098	0.057	0.096

**Table 9:** CCD-optimized variables at different levels, with experimental results.

No ofExperiment	Variable Code				Dry Weight (g_DW_/dm^3^)	Lipid Concentration (g/dm^3^)
	Z_1_	Z_2_	Z_3_	Z_4_		
1	−1	−1	−1	−1	74.6	33.0
2	1	−1	−1	−1	82.9	48.8
3	−1	1	−1	−1	75.0	49.0
4	1	1	−1	−1	82.3	45.2
5	−1	−1	1	−1	75.0	31.8
6	1	−1	1	−1	82.5	46.4
7	−1	1	1	−1	80.0	47.3
8	1	1	1	−1	83.2	44.0
9	−1	−1	−1	1	75.1	33.3
10	1	−1	−1	1	81.1	42.2
11	−1	1	−1	1	79.1	46.0
12	1	1	−1	1	84.2	49.0
13	−1	−1	1	1	75.0	47.0
14	1	−1	1	1	82.9	47.5
15	−1	1	1	1	80.4	47.8
16	1	1	1	1	82.2	46.2
17	−2	0	0	0	75.0	45.0
18	2	0	0	0	82.5	37.2
19	0	−2	0	0	82.4	45.7
20	0	2	0	0	84.5	43.7
21	0	0	−2	0	84.1	47.8
22	0	0	2	0	83.4	46.4
23	0	0	0	−2	83.9	50.1
24	0	0	0	2	83.5	48.7
25	0	0	0	0	82.7	48.8
26	0	0	0	0	81.6	47.9
27	0	0	0	0	82.1	48.0
28	0	0	0	0	81.9	48.5
29	0	0	0	0	81.0	48.0
30	0	0	0	0	80.4	47.0

**Table 10:** Values of coefficients for the second-order polynomial equation.

Variable	Dry Weight (g_DW_/dm^3^)			Lipid Concentration (g/dm^3^)		
	Estimate	*F* Value	*p*-Value	Estimate	*F* Value	*p*-Value
Z_0_	81.61			48.03		
Z_1_-Temperature	2.588	38.552	0.000	0.771	0.800	0.385
Z_2_-Glycerol level	0.896	4.621	0.048	1.688	3.835	0.069
Z_3_-Oxygen saturation	0.229	0.302	0.590	0.363	0.177	0.680
Z_4_-Yeast extract	0.154	0.137	0.717	0.446	0.268	0.612
Z_1_ Z_2_	−0.769	2.269	0.153	−2.844	7.260	0.017
Z_1_ Z_3_	−0.394	0.595	0.452	−0.856	0.658	0.430
Z_1_ Z_4_	−0.344	0.454	0.511	−0.781	0.548	0.471
Z_2_ Z_3_	0.219	0.184	0.674	−1.206	1.306	0.271
Z_2_ Z_4_	0.394	0.595	0.452	−0.406	0.148	0.706
Z_3_ Z_4_	−0.306	0.360	0.557	1.531	2.105	0.167
Z_1_^2^	−1.166	8.941	0.009	−1.991	6.098	0.026
Z_2_^2^	0.009	0.001	0.981	−1.091	1.831	0.196
Z_3_^2^	0.084	0.047	0.832	−0.491	0.370	0.552
Z_4_^2^	0.072	0.034	0.856	0.084	0.011	0.918

**Table 11:** Comparison of experimental data with the values predicted by the CCD mathematical mode.

Culture Conditions		Dry Weight (g_DW_/dm^3^)	r_DW_ (g_DW_/dm^3^xh)
Optimal values for dry biomass production	Predicted value	83.4	-
	Experimental value	84.0 ± 0.11	0.66
	Error (%)	+0.6	-
**Culture Conditions**		**Lipid Concentration (g/dm^3^)**	**r_LIP_ (g/dm^3^xh)**
Optimal values for lipid accumulation	Predicted value	54.1	-
	Experimental value	54.8 ± 0.1	0.38
	Error (%)	+1.2	-

## Data Availability

Not applicable.

## References

[1] Jariah N.F., Hassan M.A., Taufiq-Yap Y.H., Roslan A.M. (2021). Technological Advancement for Efficiency Enhancement of Biodiesel and Residual Glycerol Refining: A Mini Review. Processes.

[2] Simionescu M., Bilan Y., Zawadzki P., Wojciechowski A., Rabe M. (2021). GHG Emissions Mitigation in the European Union Based on Labor Market Changes. Energies.

[3] Khan S., Naushad M., Iqbal J., Bathula C., Sharma G. (2021). Production and harvesting of microalgae and an efficient operational approach to biofuel production for a sustainable environment. Fuel.

[4] Chandrasekhar K., Kumar S., Lee B.-D., Kim S.-H. (2020). Waste based hydrogen production for circular bioeconomy: Current status and future directions. Bioresour. Technol..

[5] Dębowski M., Zieliński M., Świca I., Kazimierowicz J. (2021). Algae Biomass as a Potential Source of Liquid Fuels. Phycology.

[6] Crosse A.J., Brady D., Zhou N., Rumbold K. (2020). Biodiesel’s trash is a biorefineries’ treasure: The use of “dirty” glycerol as an industrial fermentation substrate. World J. Microbiol. Biotechnol..

[7] Chen C.-Y., Lee M.-H., Leong Y.K., Chang J.-S., Lee D.-J. (2020). Biodiesel production from heterotrophic oleaginous microalga *Thraustochytrium* sp. BM2 with enhanced lipid accumulation using crude glycerol as alternative carbon source. Bioresour. Technol..

[8] Kujawska N., Talbierz S., Dębowski M., Kazimierowicz J., Zieliński M. (2021). Cultivation Method Effect on *Schizochytrium* sp. Biomass Growth and Docosahexaenoic Acid (DHA) Production with the Use of Waste Glycerol as a Source of Organic Carbon. Energies.

[9] Kujawska N., Talbierz S., Dębowski M., Kazimierowicz J., Zieliński M. (2021). Optimizing Docosahexaenoic Acid (DHA) Production by *Schizochytrium* sp. Grown on Waste Glycerol. Energies.

[10] Kosamia N.M., Samavi M., Uprety B.K., Rakshit S.K. (2020). Valorization of Biodiesel Byproduct Crude Glycerol for the Production of Bioenergy and Biochemicals. Catalysts.

[11] Ortiz Ruiz A. (2021). Microalgae-Based Wastewater Treatment Systems at Demonstrative Scale: Gravity Harvesting and Thickening of Biomass, and Advanced Design of Bioreactors. Ph.D. Thesis.

[12] González-Balderas R.M., Felix M., Bengoechea C., Orta Ledesma M.T., Guerrero A., Velasquez-Orta S.B. (2021). Development of composites based on residual microalgae biomass cultivated in wastewater. Eur. Polym. J..

[13] Borowiak D., Lenartowicz P., Grzebyk M., Wiśniewski M., Lipok J., Kafarski P. (2021). Novel, automated, semi-industrial modular photobioreactor system for cultivation of demanding microalgae that produce fine chemicals—The next story of *H. pluvialis* and astaxanthin. Algal Res..

[14] Shekh A., Sharma A., Schenk P.M., Kumar G., Mudliar S. (2021). Microalgae cultivation: Photobioreactors, CO_2_ utilization, and value-added products of industrial importance. J. Chem. Technol. Biotechnol..

[15] Kumar V., Sharma N., Jaiswal K.K., Vlaskin M.S., Nanda M., Tripathi M.K., Kumar S. (2021). Microalgae with a truncated light-harvesting antenna to maximize photosynthetic efficiency and biomass productivity: Recent advances and current challenges. Process Biochem..

[16] Sharamo F.F., Shimelis H., OlaOlorun B.M., Korir H., Indetie A.H., Mashilo J. (2021). Determining ethyl methane sulfonate-mediated (EMS) mutagenesis protocol for inducing high biomass yield in fodder barley (‘*Hordeum vulgare*’ L.). Aust. J. Crop Sci..

[17] Watford S., Warrington S.J. (2017). Bacterial DNA Mutations. StatPearls [Internet].

[18] Siddique M.I., Back S., Lee J.-H., Jo J., Jang S., Han K., Venkatesh J., Kwon J.-K., Jo Y.D., Kang B.-C. (2020). Development and Characterization of an Ethyl Methane Sulfonate (EMS) Induced Mutant Population in *Capsicum annuum* L.. Plants.

[19] Khalil F., Naiyan X., Tayyab M., Pinghua C. (2018). Screening of EMS-Induced Drought-Tolerant Sugarcane Mutants Employing Physiological, Molecular and Enzymatic Approaches. Agronomy.

[20] Smith-Baedorf H.D. (2012). Microalgae for the Biochemical Conversion of CO_2_ and Production of Biodiesel. Ph.D. Thesis.

[21] Murbach T.S., Glávits R., Endres J.R., Hirka G., Vértesi A., Béres E., Szakonyiné I.P. (2018). A toxicological evaluation of *Chlamydomonas reinhardtii*, a green algae. Int. J. Toxicol..

[22] Kawaroe M., Sudrajat A.O., Hwangbo J., Augustine D. (2015). Chemical mutagenesis of microalgae *Nannochloropsis* sp. using ems (ethyl methanesulfonate). Br. J. Appl. Sci. Technol..

[23] Huang G.-H., Chen G., Chen F. (2009). Rapid screening method for lipid production in alga based on Nile red fluorescence. Biomass Bioenergy.

[24] Cohen Z., Shiran D., Khozin I., Heimer Y.M. (1997). Fatty acid unsaturation in the red alga *Porphyridium cruentum*. Is the methylene interrupted nature of polyunsaturated fatty acids an intrinsic property of the desaturases?. Biochim. Et Biophys. Acta (BBA)–Lipids Lipid Metab..

[25] Zhang Y., He M., Zou S., Fei C., Yan Y., Zheng S., Rajper A.A., Wang C. (2016). Breeding of high biomass and lipid producing *Desmodesmus* sp. by Ethylmethane sulfonate-induced mutation. Bioresour. Technol..

[26] Tanadul O., Noochanong W., Jirakranwong P., Chanprame S. (2018). EMS-induced mutation followed by quizalofop-screening increased lipid productivity in *Chlorella* sp.. Bioprocess Biosyst. Eng..

[27] Dinesh Kumar S., Sojin K., Santhanam P., Dhanalakshmi B., Latha S., Park M.S., Kim M.-K. (2018). Triggering of fatty acids on *Tetraselmis* sp. by ethyl methanesulfonate mutagenic treatment. Bioresour. Technol. Rep..

[28] Kawaroe M., Prartono T., Hwangbo J., Sunuddin A., Augustine D., Gustina A.S. (2015). Effect of ethyl methane sulfonate (EMS) on cell size, fatty acid content, growth rate, and antioxidant activities of microalgae *Dunaliella* sp.. AACL Bioflux.

[29] Sarayloo E., Tardu M., Unlu Y.S., Simsek S., Cevahir G., Erkey C., Kavakli I.H. (2017). Understanding lipid metabolism in high-lipid-producing *Chlorella vulgaris* mutants at the genome-wide level. Algal Res..

[30] Yokochi T., Honda D., Higashihara T., Nakahara T. (1998). Optimization of docosahexaenoic acid production by *Schizochytrium limacinum* SR21. Appl. Microbiol. Biotechnol..

[31] Kong W., Yang S., Wang H., Huo H., Guo B., Liu N., Zhang A., Niu S. (2020). Regulation of biomass, pigments, and lipid production by *Chlorella vulgaris* 31 through controlling trophic modes and carbon sources. J. Appl. Phycol..

[32] Capa-Robles W., García-Mendoza E., Paniagua-Michel J.d.J. (2021). Enhanced β-carotene and Biomass Production by Induced Mixotrophy in *Dunaliella salina* across a Combined Strategy of Glycerol, Salinity, and Light. Metabolites.

[33] Sohrabi D., Jazini M.H., Shariati M. (2019). Mixotrophic cultivation of *Dunaliella salina* on crude glycerol obtained from calcinated fatty acid production process. Russ. J. Mar. Biol..

[34] Rattanapoltee P., Dujjanutat P., Muanruksa P., Kaewkannetra P. (2021). Biocircular platform for third generation biodiesel production: Batch/fed batch mixotrophic cultivations of microalgae using glycerol waste as a carbon source. Biochem. Eng. J..

[35] Pyle D.J., Garcia R.A., Wen Z. (2008). Producing docosahexaenoic acid (DHA)-rich algae from biodiesel-derived crude glycerol: Effects of impurities on DHA production and algal biomass composition. J. Agric. Food Chem..

[36] Chang G., Gao N., Tian G., Wu Q., Chang M., Wang X. (2013). Improvement of docosahexaenoic acid production on glycerol by *Schizochytrium* sp. S31 with constantly high oxygen transfer coefficient. Bioresour. Technol..

[37] Huang T.Y., Lu W.C., Chu I.M. (2012). A fermentation strategy for producing docosahexaenoic acid in *Aurantiochytrium limacinum* SR21 and increasing C22:6 proportions in total fatty acid. Bioresour. Technol..

[38] Yeh K.-L., Chang J.-S. (2012). Effects of cultivation conditions and media composition on cell growth and lipid productivity of indigenous microalga *Chlorella vulgaris* ESP-31. Bioresour. Technol..

[39] Hu Q., Sommerfeld M., Jarvis E., Ghirardi M., Posewitz M., Seibert M., Darzins A. (2008). Microalgal triacylglycerols as feedstocks for biofuel production: Perspectives and advances. Plant J..

[40] Chi Z., Liu Y., Frear C., Chen S. (2009). Study of a two-stage growth of DHA-producing marine algae *Schizochytrium limacinum* SR21 with shifting dissolved oxygen level. Appl. Microbiol. Biotechnol..

[41] Nagappan S., Devendran S., Tsai P.-C., Dahms H.-U., Ponnusamy V.K. (2019). Potential of two-stage cultivation in microalgae biofuel production. Fuel.

